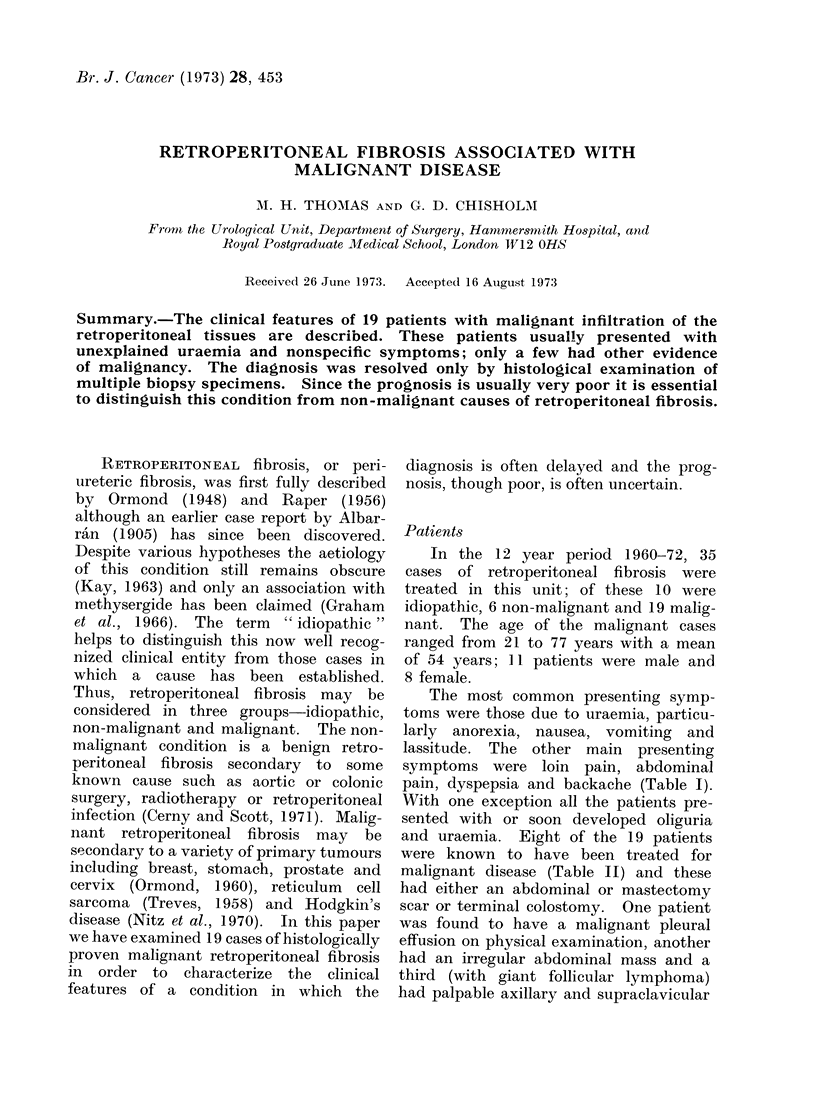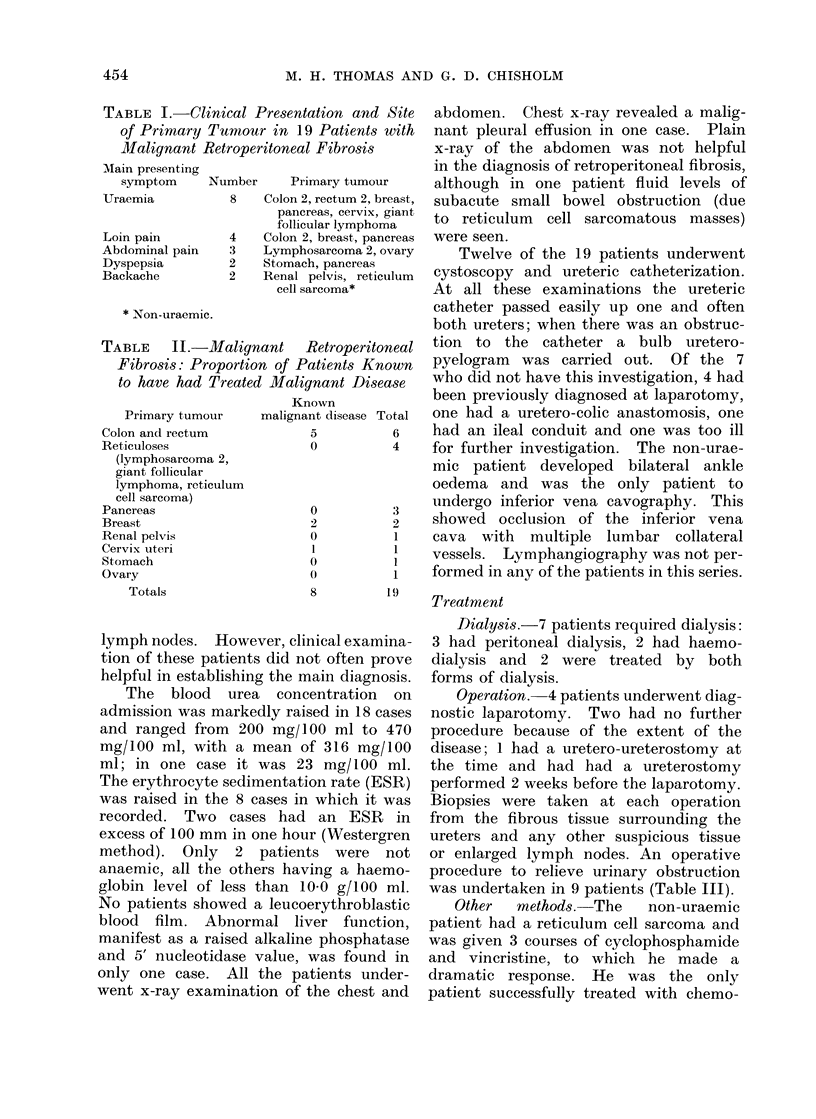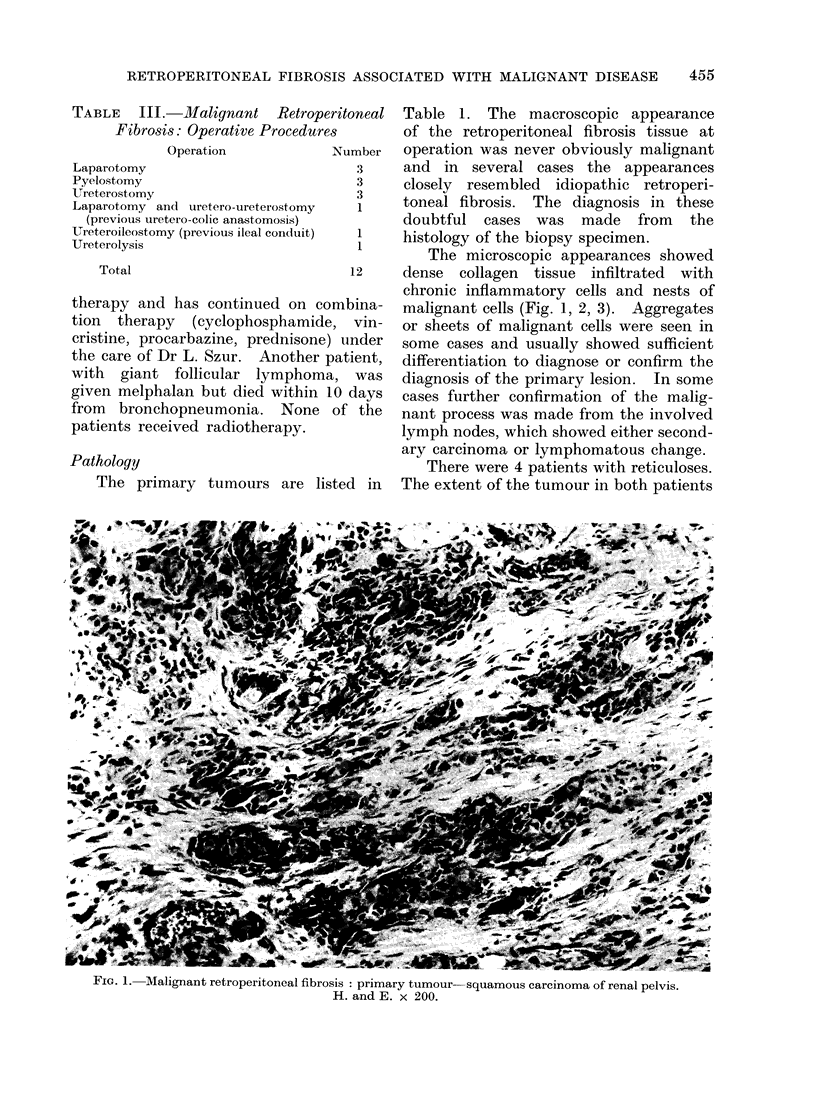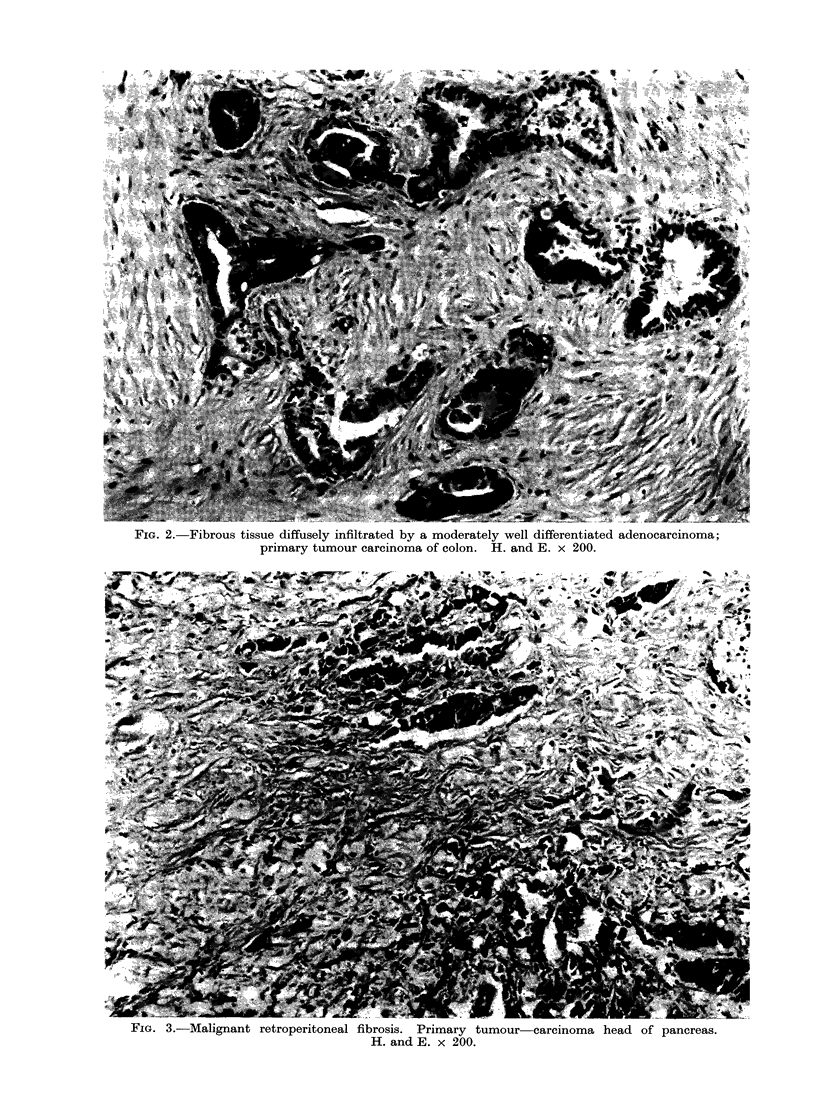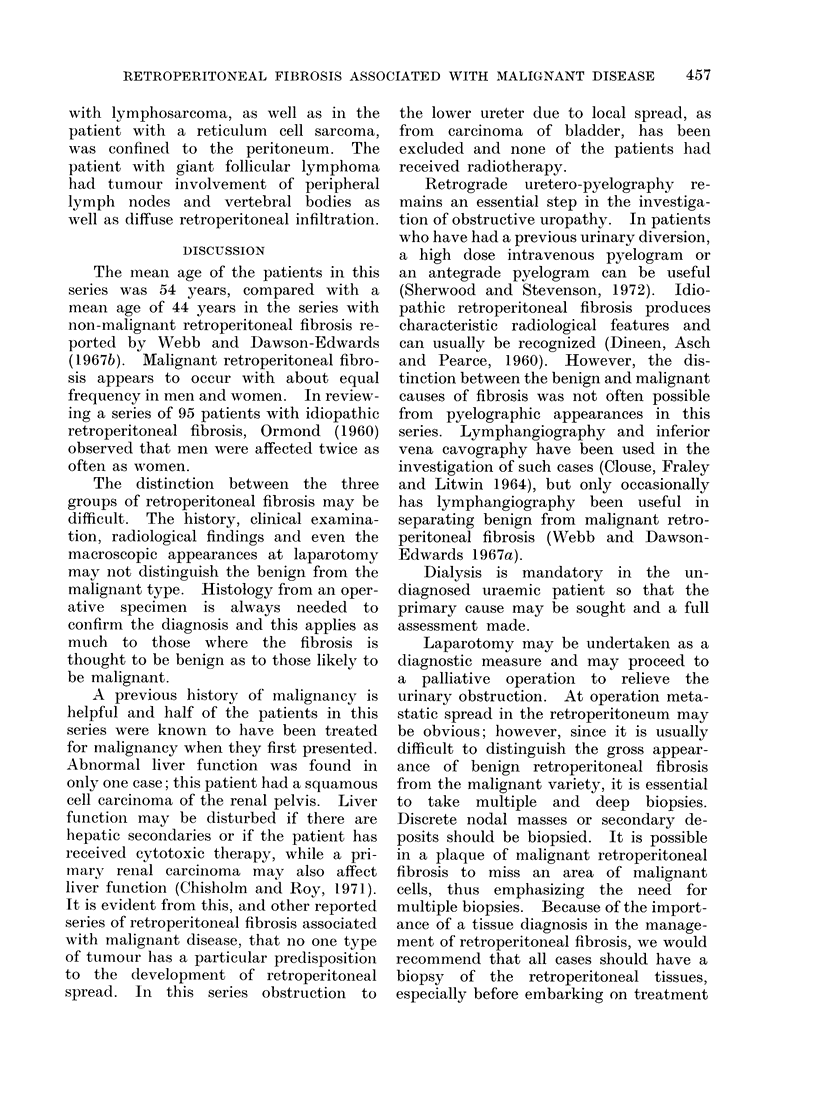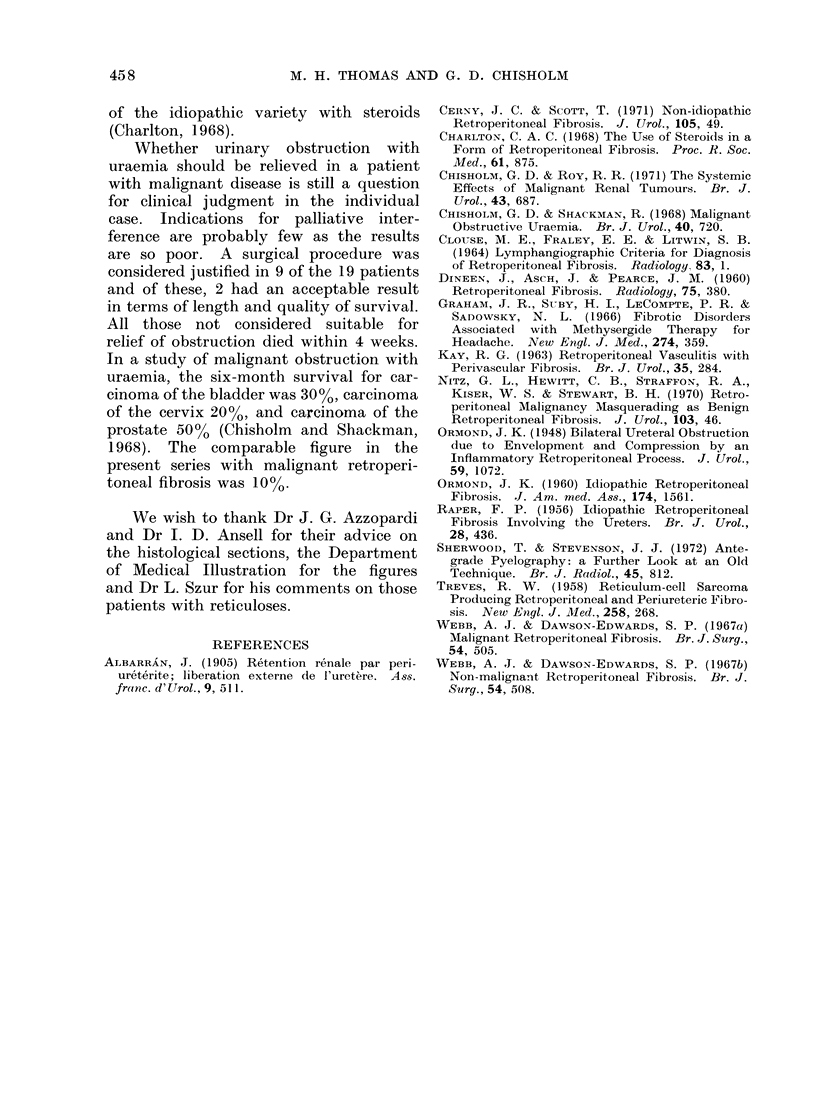# Retroperitoneal Fibrosis Associated with Malignant Disease

**DOI:** 10.1038/bjc.1973.171

**Published:** 1973-11

**Authors:** M. H. Thomas, G. D. Chisholm

## Abstract

**Images:**


*Br. J. Cancer* (1973) **28**, 453

### RETROPERITONEAL FIBROSIS ASSOCIATED WITH MALIGNANT DISEASE

M. H. THOMAS AND G. D. CHISHOLM

*From the Urological Unit, Department of Surgery, Hammersmith Hospital, and Royal Postgraduate Medical School, London W12 0HS*

Received 26 June 1973. Accepted 16 August 1973

**Summary.—The clinical features of 19 patients with malignant infiltration of the retroperitoneal tissues are described. These patients usually presented with unexplained uraemia and nonspecific symptoms; only a few had other evidence of malignancy. The diagnosis was resolved only by histological examination of multiple biopsy specimens. Since the prognosis is usually very poor it is essential to distinguish this condition from non-malignant causes of retroperitoneal fibrosis.**

RETROPERITONEAL fibrosis, or periureteric fibrosis, was first fully described by Ormond (1948) and Raper (1956) although an earlier case report by Albarrán (1905) has since been discovered. Despite various hypotheses the aetiology of this condition still remains obscure (Kay, 1963) and only an association with methysergide has been claimed (Graham *et al.*, 1966). The term "idiopathic" helps to distinguish this now well recognized clinical entity from those cases in which a cause has been established. Thus, retroperitoneal fibrosis may be considered in three groups—idiopathic, non-malignant and malignant. The non-malignant condition is a benign retroperitoneal fibrosis secondary to some known cause such as aortic or colonic surgery, radiotherapy or retroperitoneal infection (Cerny and Scott, 1971). Malignant retroperitoneal fibrosis may be secondary to a variety of primary tumours including breast, stomach, prostate and cervix (Ormond, 1960), reticulum cell sarcoma (Treves, 1958) and Hodgkin's disease (Nitz *et al.*, 1970). In this paper we have examined 19 cases of histologically proven malignant retroperitoneal fibrosis in order to characterize the clinical features of a condition in which the diagnosis is often delayed and the prognosis, though poor, is often uncertain.

### *Patients*

In the 12 year period 1960–72, 35 cases of retroperitoneal fibrosis were treated in this unit; of these 10 were idiopathic, 6 non-malignant and 19 malignant. The age of the malignant cases ranged from 21 to 77 years with a mean of 54 years; 11 patients were male and 8 female.

The most common presenting symptoms were those due to uraemia, particularly anorexia, nausea, vomiting and lassitude. The other main presenting symptoms were loin pain, abdominal pain, dyspepsia and backache (Table I). With one exception all the patients presented with or soon developed oliguria and uraemia. Eight of the 19 patients were known to have been treated for malignant disease (Table II) and these had either an abdominal or mastectomy scar or terminal colostomy. One patient was found to have a malignant pleural effusion on physical examination, another had an irregular abdominal mass and a third (with giant follicular lymphoma) had palpable axillary and supraclavicular

TABLE I.—*Clinical Presentation and Site of Primary Tumour in 19 Patients with Malignant Retroperitoneal Fibrosis*

| Main presenting symptom | Number | Primary tumour |
|---|---|---|
| Uraemia | 8 | Colon 2, rectum 2, breast, pancreas, cervix, giant follicular lymphoma |
| Loin pain | 4 | Colon 2, breast, pancreas |
| Abdominal pain | 3 | Lymphosarcoma 2, ovary |
| Dyspepsia | 2 | Stomach, pancreas |
| Backache | 2 | Renal pelvis, reticulum cell sarcoma* |

* Non-uraemic.

TABLE II.—*Malignant Retroperitoneal Fibrosis: Proportion of Patients Known to have had Treated Malignant Disease*

| Primary tumour | Known malignant disease | Total |
|---|---|---|
| Colon and rectum | 5 | 6 |
| Reticuloses (lymphosarcoma 2, giant follicular lymphoma, reticulum cell sarcoma) | 0 | 4 |
| Pancreas | 0 | 3 |
| Breast | 2 | 2 |
| Renal pelvis | 0 | 1 |
| Cervix uteri | 1 | 1 |
| Stomach | 0 | 1 |
| Ovary | 0 | 1 |
| Totals | 8 | 19 |

lymph nodes. However, clinical examination of these patients did not often prove helpful in establishing the main diagnosis.

The blood urea concentration on admission was markedly raised in 18 cases and ranged from 200 mg/100 ml to 470 mg/100 ml, with a mean of 316 mg/100 ml; in one case it was 23 mg/100 ml. The erythrocyte sedimentation rate (ESR) was raised in the 8 cases in which it was recorded. Two cases had an ESR in excess of 100 mm in one hour (Westergren method). Only 2 patients were not anaemic, all the others having a haemoglobin level of less than 10·0 g/100 ml. No patients showed a leucoerythroblastic blood film. Abnormal liver function, manifest as a raised alkaline phosphatase and 5′ nucleotidase value, was found in only one case. All the patients underwent x-ray examination of the chest and abdomen. Chest x-ray revealed a malignant pleural effusion in one case. Plain x-ray of the abdomen was not helpful in the diagnosis of retroperitoneal fibrosis, although in one patient fluid levels of subacute small bowel obstruction (due to reticulum cell sarcomatous masses) were seen.

Twelve of the 19 patients underwent cystoscopy and ureteric catheterization. At all these examinations the ureteric catheter passed easily up one and often both ureters; when there was an obstruction to the catheter a bulb uretero-pyelogram was carried out. Of the 7 who did not have this investigation, 4 had been previously diagnosed at laparotomy, one had a uretero-colic anastomosis, one had an ileal conduit and one was too ill for further investigation. The non-uraemic patient developed bilateral ankle oedema and was the only patient to undergo inferior vena cavography. This showed occlusion of the inferior vena cava with multiple lumbar collateral vessels. Lymphangiography was not performed in any of the patients in this series.

*Treatment*

*Dialysis*.—7 patients required dialysis: 3 had peritoneal dialysis, 2 had haemodialysis and 2 were treated by both forms of dialysis.

*Operation*.—4 patients underwent diagnostic laparotomy. Two had no further procedure because of the extent of the disease; 1 had a uretero-ureterostomy at the time and had had a ureterostomy performed 2 weeks before the laparotomy. Biopsies were taken at each operation from the fibrous tissue surrounding the ureters and any other suspicious tissue or enlarged lymph nodes. An operative procedure to relieve urinary obstruction was undertaken in 9 patients (Table III).

*Other methods*.—The non-uraemic patient had a reticulum cell sarcoma and was given 3 courses of cyclophosphamide and vincristine, to which he made a dramatic response. He was the only patient successfully treated with chemo-

TABLE III.—*Malignant Retroperitoneal Fibrosis: Operative Procedures*

| Operation | Number |
|---|---|
| Laparotomy | 3 |
| Pyelostomy | 3 |
| Ureterostomy | 3 |
| Laparotomy and uretero-ureterostomy (previous uretero-colic anastomosis) | 1 |
| Ureteroileostomy (previous ileal conduit) | 1 |
| Ureterolysis | 1 |
| Total | 12 |

therapy and has continued on combination therapy (cyclophosphamide, vincristine, procarbazine, prednisone) under the care of Dr L. Szur. Another patient, with giant follicular lymphoma, was given melphalan but died within 10 days from bronchopneumonia. None of the patients received radiotherapy.

*Pathology*

The primary tumours are listed in Table 1. The macroscopic appearance of the retroperitoneal fibrosis tissue at operation was never obviously malignant and in several cases the appearances closely resembled idiopathic retroperitoneal fibrosis. The diagnosis in these doubtful cases was made from the histology of the biopsy specimen.

The microscopic appearances showed dense collagen tissue infiltrated with chronic inflammatory cells and nests of malignant cells (Fig. 1, 2, 3). Aggregates or sheets of malignant cells were seen in some cases and usually showed sufficient differentiation to diagnose or confirm the diagnosis of the primary lesion. In some cases further confirmation of the malignant process was made from the involved lymph nodes, which showed either secondary carcinoma or lymphomatous change.

There were 4 patients with reticuloses. The extent of the tumour in both patients

FIG. 1.—Malignant retroperitoneal fibrosis : primary tumour—squamous carcinoma of renal pelvis. H. and E. × 200.

FIG. 2.—Fibrous tissue diffusely infiltrated by a moderately well differentiated adenocarcinoma; primary tumour carcinoma of colon. H. and E. × 200.

FIG. 3.—Malignant retroperitoneal fibrosis. Primary tumour—carcinoma head of pancreas. H. and E. × 200.

with lymphosarcoma, as well as in the patient with a reticulum cell sarcoma, was confined to the peritoneum. The patient with giant follicular lymphoma had tumour involvement of peripheral lymph nodes and vertebral bodies as well as diffuse retroperitoneal infiltration.

### DISCUSSION

The mean age of the patients in this series was 54 years, compared with a mean age of 44 years in the series with non-malignant retroperitoneal fibrosis reported by Webb and Dawson-Edwards (1967*b*). Malignant retroperitoneal fibrosis appears to occur with about equal frequency in men and women. In reviewing a series of 95 patients with idiopathic retroperitoneal fibrosis, Ormond (1960) observed that men were affected twice as often as women.

The distinction between the three groups of retroperitoneal fibrosis may be difficult. The history, clinical examination, radiological findings and even the macroscopic appearances at laparotomy may not distinguish the benign from the malignant type. Histology from an operative specimen is always needed to confirm the diagnosis and this applies as much to those where the fibrosis is thought to be benign as to those likely to be malignant.

A previous history of malignancy is helpful and half of the patients in this series were known to have been treated for malignancy when they first presented. Abnormal liver function was found in only one case; this patient had a squamous cell carcinoma of the renal pelvis. Liver function may be disturbed if there are hepatic secondaries or if the patient has received cytotoxic therapy, while a primary renal carcinoma may also affect liver function (Chisholm and Roy, 1971). It is evident from this, and other reported series of retroperitoneal fibrosis associated with malignant disease, that no one type of tumour has a particular predisposition to the development of retroperitoneal spread. In this series obstruction to the lower ureter due to local spread, as from carcinoma of bladder, has been excluded and none of the patients had received radiotherapy.

Retrograde uretero-pyelography remains an essential step in the investigation of obstructive uropathy. In patients who have had a previous urinary diversion, a high dose intravenous pyelogram or an antegrade pyelogram can be useful (Sherwood and Stevenson, 1972). Idiopathic retroperitoneal fibrosis produces characteristic radiological features and can usually be recognized (Dineen, Asch and Pearce, 1960). However, the distinction between the benign and malignant causes of fibrosis was not often possible from pyelographic appearances in this series. Lymphangiography and inferior vena cavography have been used in the investigation of such cases (Clouse, Fraley and Litwin 1964), but only occasionally has lymphangiography been useful in separating benign from malignant retroperitoneal fibrosis (Webb and Dawson-Edwards 1967*a*).

Dialysis is mandatory in the undiagnosed uraemic patient so that the primary cause may be sought and a full assessment made.

Laparotomy may be undertaken as a diagnostic measure and may proceed to a palliative operation to relieve the urinary obstruction. At operation metastatic spread in the retroperitoneum may be obvious; however, since it is usually difficult to distinguish the gross appearance of benign retroperitoneal fibrosis from the malignant variety, it is essential to take multiple and deep biopsies. Discrete nodal masses or secondary deposits should be biopsied. It is possible in a plaque of malignant retroperitoneal fibrosis to miss an area of malignant cells, thus emphasizing the need for multiple biopsies. Because of the importance of a tissue diagnosis in the management of retroperitoneal fibrosis, we would recommend that all cases should have a biopsy of the retroperitoneal tissues, especially before embarking on treatment of the idiopathic variety with steroids (Charlton, 1968).

Whether urinary obstruction with uraemia should be relieved in a patient with malignant disease is still a question for clinical judgment in the individual case. Indications for palliative interference are probably few as the results are so poor. A surgical procedure was considered justified in 9 of the 19 patients and of these, 2 had an acceptable result in terms of length and quality of survival. All those not considered suitable for relief of obstruction died within 4 weeks. In a study of malignant obstruction with uraemia, the six-month survival for carcinoma of the bladder was 30%, carcinoma of the cervix 20%, and carcinoma of the prostate 50% (Chisholm and Shackman, 1968). The comparable figure in the present series with malignant retroperitoneal fibrosis was 10%.

We wish to thank Dr J. G. Azzopardi and Dr I. D. Ansell for their advice on the histological sections, the Department of Medical Illustration for the figures and Dr L. Szur for his comments on those patients with reticuloses.

### REFERENCES

Albarrán, J. (1905) Rétention rénale par periurétérite; liberation externe de l'uretère. *Ass. franc. d'Urol.*, **9**, 511.

Cerny, J. C. & Scott, T. (1971) Non-idiopathic Retroperitoneal Fibrosis. *J. Urol.*, **105**, 49.

Charlton, C. A. C. (1968) The Use of Steroids in a Form of Retroperitoneal Fibrosis. *Proc. R. Soc. Med.*, **61**, 875.

Chisholm, G. D. & Roy, R. R. (1971) The Systemic Effects of Malignant Renal Tumours. *Br. J. Urol.*, **43**, 687.

Chisholm, G. D. & Shackman, R. (1968) Malignant Obstructive Uraemia. *Br. J. Urol.*, **40**, 720.

Clouse, M. E., Fraley, E. E. & Litwin, S. B. (1964) Lymphangiographic Criteria for Diagnosis of Retroperitoneal Fibrosis. *Radiology*, **83**, 1.

Dineen, J., Asch, J. & Pearce, J. M. (1960) Retroperitoneal Fibrosis. *Radiology*, **75**, 380.

Graham, J. R., Suby, H. I., LeCompte, P. R. & Sadowsky, N. L. (1966) Fibrotic Disorders Associated with Methysergide Therapy for Headache. *New Engl. J. Med.*, **274**, 359.

Kay, R. G. (1963) Retroperitoneal Vasculitis with Perivascular Fibrosis. *Br. J. Urol.*, **35**, 284.

Nitz, G. L., Hewitt, C. B., Straffon, R. A., Kiser, W. S. & Stewart, B. H. (1970) Retroperitoneal Malignancy Masquerading as Benign Retroperitoneal Fibrosis. *J. Urol.*, **103**, 46.

Ormond, J. K. (1948) Bilateral Ureteral Obstruction due to Envelopment and Compression by an Inflammatory Retroperitoneal Process. *J. Urol.*, **59**, 1072.

Ormond, J. K. (1960) Idiopathic Retroperitoneal Fibrosis. *J. Am. med. Ass.*, **174**, 1561.

Raper, F. P. (1956) Idiopathic Retroperitoneal Fibrosis Involving the Ureters. *Br. J. Urol.*, **28**, 436.

Sherwood, T. & Stevenson, J. J. (1972) Antegrade Pyelography: a Further Look at an Old Technique. *Br. J. Radiol.*, **45**, 812.

Treves, R. W. (1958) Reticulum-cell Sarcoma Producing Retroperitoneal and Periureteric Fibrosis. *New Engl. J. Med.*, **258**, 268.

Webb, A. J. & Dawson-Edwards, S. P. (1967*a*) Malignant Retroperitoneal Fibrosis. *Br. J. Surg.*, **54**, 505.

Webb, A. J. & Dawson-Edwards, S. P. (1967*b*) Non-malignant Retroperitoneal Fibrosis. *Br. J. Surg.*, **54**, 508.